# Dual roles of cellular communication network factor 6 (CCN6) in the invasion and metastasis of oral cancer cells to bone via binding to BMP2 and RANKL

**DOI:** 10.1093/carcin/bgad057

**Published:** 2023-08-17

**Authors:** Hiroaki Hochi, Satoshi Kubota, Masaharu Takigawa, Takashi Nishida

**Affiliations:** Department of Biochemistry and Molecular Dentistry, Okayama University Graduate School of Medicine, Dentistry, and Pharmaceutical Sciences, Okayama 700-8525, Japan; Department of Biochemistry and Molecular Dentistry, Okayama University Graduate School of Medicine, Dentistry, and Pharmaceutical Sciences, Okayama 700-8525, Japan; Advanced Research Center for Oral and Craniofacial Sciences, Okayama University Faculty of Medicine, Dentistry, and Pharmaceutical Sciences, Okayama 700-8525, Japan; Department of Biochemistry and Molecular Dentistry, Okayama University Graduate School of Medicine, Dentistry, and Pharmaceutical Sciences, Okayama 700-8525, Japan

## Abstract

The acquisition of motility via epithelial–mesenchymal transition (EMT) and osteoclast induction are essential for the invasion and metastasis of oral squamous cell carcinoma (OSCC) to bone. However, the molecule suppressing both EMT and osteoclastogenesis is still unknown. In this study, we found that cellular communication network factor 6 (CCN6) was less produced in a human OSCC cell line, HSC-3 with mesenchymal phenotype, than in HSC-2 cells without it. Notably, CCN6 interacted with bone morphogenetic protein 2 (BMP2) and suppressed the cell migration of HSC-3 cells stimulated by BMP2. Moreover, knockdown of CCN6 in HSC-2 cells led to the promotion of EMT and enhanced the effect of transforming growth factor-β (TGF-β) on the promotion of EMT. Furthermore, CCN6 combined with BMP2 suppressed EMT. These results suggest that CCN6 strongly suppresses EMT in cooperation with BMP2 and TGF-β. Interestingly, CCN6 combined with BMP2 increased the gene expression of receptor activator of nuclear factor-κB ligand (RANKL) in HSC-2 and HSC-3 cells. Additionally, CCN6 interacted with RANKL, and CCN6 combined with RANKL suppressed RANKL-induced osteoclast formation. In metastatic lesions, increasing BMP2 due to the bone destruction led to interference with binding of CCN6 to RANKL, which results in the promotion of bone metastasis of OSCC cells due to continuous osteoclastogenesis. These findings suggest that CCN6 plays dual roles in the suppression of EMT and in the promotion of bone destruction of OSCC in primary and metastatic lesions, respectively, through cooperation with BMP2 and interference with RANKL.

## Teaser

We propose the dual roles of CCN6 in OSCC cells. In primary lesions, CCN6 combined with BMP2 inhibits EMT. In metastatic lesions, BMP2-bound CCN6 promotes MET and RANKL expression, resulting in the promotion of bone metastasis.

## Graphical Abstract

**Graphical Abstract:**
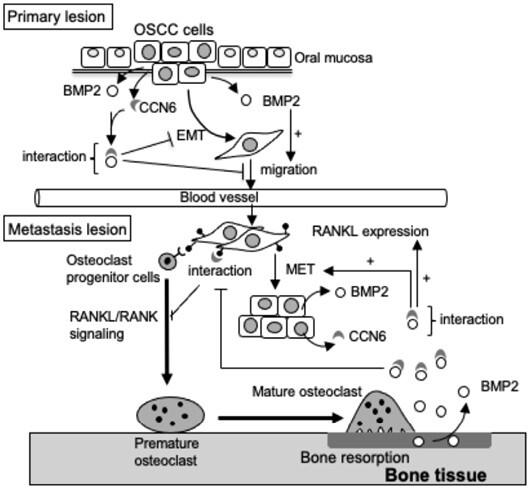

## Introduction

Oral cancers are malignant tumors that develop in the oral cavity, including tongue, lip and gingiva, and more than 90% of their histopathological findings are oral squamous cell carcinoma (OSCC) (1). In the USA, oral cancer incidence is approximately 50,000 cases annually, and it has been reported that 20% of these cases die per year (2). On the other hand, the incidence in Japan is approximately 20,000 cases annually, and the rate of death is more than 35%, with the mortality rate increasing year by year (3).

Cancer cells acquire phenotypic changes, such as mobility, stress resistance, immune tolerance and so on, through an epithelial–mesenchymal transition (EMT) induced by several factors and metastasize to distinct organs. This is considered to be one of the major causes of the mortality by cancer cells (1). Therefore, suppression of EMT in OSCCs leads to their attenuated invasion and metastasis to another organ. In particular, the destruction of jawbone caused by invading OSCCs is associated with poor prognosis (4). Of note, EMT, which is associated with motility acquisition, and osteoclast formation, which is associated with bone tissue destruction, are suspected to play major roles in the invasion of OSCCs in bone tissues (4). However, the mechanisms of OSCC bone invasion are still unknown, compared with those of breast cancer and prostate cancer cells, which tend to metastasize to the bone tissues. Previously, in order to investigate the mechanisms of OSCC invasion to multiple organs, Momose *et al.* established three different OSCC-derived cell lines, HSC-2, HSC-3 and HSC-4, from neck lymph node metastases of patients with OSCC developed in tongues (5). They inoculated these tumor cells into nude mice subcutaneously and reported the differences in the histological finding (5). Namely, HSC-2 and HSC-4 cells produced keratin and grew to compress the surrounding tissues, forming a capsule, whereas HSC-3 cells grew diffusely and invaded the subcutaneous tissues and a part of the muscle layer (5). These findings indicate that HSC-2 and HSC-4 cells are highly differentiated tumor cells, and HSC-3 cells are lower differentiated ones. Based on these results, we considered that HSC-2 and HSC-3 cells could be useful in clarifying the mechanisms of bone metastasis by comparative analyses.

Cellular communication network factors (CCNs) are matricellular proteins that govern the interconnection among several signaling pathways, such as bone morphogenetic proteins (BMPs) and transforming growth factor-β (TGF-β) signaling pathways (6). The CCN family consists of six distinct proteins, which are CCN1/cysteine-rich 61 (CYR61), CCN2/connective tissue growth factor (CTGF), CCN3/nephroblastoma overexpressed (NOV), CCN4/Wnt-inducible secreted protein 1 (WISP1), CCN5/WISP2 and CCN6/WISP3 (6). These proteins have four highly conserved module structures, except for CCN5 lacking the C-terminal module, and they coordinate various cellular functions through binding to various biological molecules, such as growth factors, cytokines and extracellular proteins, with their four modules (6). Therefore, CCN proteins are involved in multiple physiological and pathological cellular functions, such as angiogenesis, cartilage development, osteoclastogenesis and carcinogenesis (6,7). In particular, in terms of OSCC growth and EMT of tumor cells, similar functions or opposite roles have been reported among CCN family proteins (8). Namely, CCN1 promotes both OSCC growth and EMT of laryngeal squamous cell carcinoma (8,9), and similar functions have also been reported for CCN4 (8,10,11). In contrast, CCN2 has been variously reported to either promote or suppress OSCC growth (8,12,13), which remains controversial. On the other hand, it has been reported that CCN3 promotes EMT in prostate and pancreatic cancer cells (8,14,15), whereas CCN5 suppresses EMT in many types of cancer cells (16–18). Furthermore, CCN6 has been reported to suppress EMT in breast (19,20) and liver cancer cells (21). In light of a retrospective analysis of the literature, we expected a molecule that controls not only EMT but also another factor regulating bone metastasis to be among the six members of CCN family proteins. In this study, we explored the CCN family proteins to identify a molecule regulating both EMT and osteoclast formation and investigated its roles by means of comparative analyses with HSC-2 cells, which have epithelial phenotypes, and HSC-3 cells, which have both epithelial and mesenchymal phenotypes.

## Materials and methods

### Materials

Cell culture dishes and multi-well plates for adherent cells were purchased from Becton Dickinson Biosciences (Bedford, MA, USA), Thermo Scientific (Waltham, MA, USA) and TrueLine (Nippon Genetics Co. Ltd., Tokyo, Japan), and non-tissue culture-treated multi-well plates were from Corning (Bedford, MA, USA). Dulbecco’s modified Eagle’s medium (DMEM) and α-modified Eagle’s medium (αMEM) were purchased from Nissui Pharmaceutical Co. Ltd. (Tokyo, Japan), and ICN Biomedicals (Aurora, OH, USA), respectively, and fetal bovine serum (FBS) was obtained from Nichirei Bioscience (Tokyo, Japan). Recombinant human CCN6 (rCCN6) and porcine TGF-β1 were purchased from PerpoTech (Rocky Hill, NJ, USA) and R&D systems (Minneapolis, MN, USA), respectively, and recombinant human BMP2 (rBMP2) was kindly provided by Osteopharma (Osaka, Japan). Both CCN6 siRNA (sc-39339) and negative control siRNA (sc-37007) were purchased from Santa Cruz Biotechnology (Santa Cruz, CA, USA). Recombinant glutathione *S*-transferase (GST)-fused receptor activator of nuclear factor-κB ligand (RANKL) was purified as described previously (22).

### Cell cultures

Human OSCC cell lines, HSC-2 (5) and HSC-3 (5), and human epidermoid carcinoma cell line, A431 that were authenticated by bacterial testing, mycoplasma screening and short tandem repeat analysis were obtained from Japanese Collection of Research Bioresources (JCRB) cell bank before 2002. These cell lines were inoculated at a density of 2 × 10^4^ cells/cm^2^ into culture dishes and multi-well plates containing DMEM supplemented with 10% FBS and were cultured at 37°C in a humidified atmosphere with 5% CO_2_. Murine macrophage-like cell line RAW264.7 was obtained from the American Type Culture Collection (ATCC) in 2006, and the RAW264.7 cells were inoculated at a density of 1 × 10^4^ cells/cm^2^ into culture dishes and multi-well plates containing αMEM supplemented with 10% FBS and were cultured at 37°C in a humidified atmosphere with 5% CO_2_ (23). RAW264.7 cells were authenticated by ability of osteoclastogenesis.

### Cell proliferation assay

Cell proliferation was evaluated by counting the cell numbers of HSC-2 and HSC-3 cells. Briefly, both HSC-2 and HSC-3 cells were inoculated in 35 mm dishes at a density of 1 × 10^4^ cells/dish (day 0) and were cultured in DMEM containing 10% FBS for 10 days. We counted the cell numbers at days 0, 1, 3, 7 and 10 from starting cell culture.

### Cell migration assay

Cell migration assay of HSC-3 cells treated with BMP2, CCN6 or both was performed using a modified Boyden chamber method. Briefly, CHEMOTAXICELL chambers with polycarbonate filters (pore size: 8-µm diameter, KURABO, Osaka, Japan) were placed in the wells of a 24-well plate containing 0.3 ml of DMEM with 10% FBS. Cell suspension of HSC-3 cells at a density of 1 × 10^4^ cells per 0.2 ml of DMEM with 10% FBS was put into upper compartments of the chambers, and 4 h later, the cells in the chambers were treated with BMP2, CCN6 or BMP2 combined with CCN6. After 20 h, the cells migrated outside through the filters were fixed with 4% formaldehyde for 30 min and stained with 4ʹ,6-diamidino-2-phenylindole. Then, non-migrated cells inside the chambers were wiped away with a cotton swab, and the cells on the outside of the filters stained with 4ʹ,6-diamidino-2-phenylindole were counted using ImageJ.

### Quantitative reverse-transcription–PCR analysis

Total RNAs from HSC-2 and HSC-3 cells were isolated by ISOGEN reagent (Nippon Gene, Tokyo, Japan). First-strand cDNA was synthesized from 1 µg total RNA using a PrimerScript™ RT reagent kit (no. RR037A, Takara Shuzo, Tokyo, Japan), and subsequent quantitative polymerase chain reaction (PCR) analysis was performed using a SYBR® Green Real-Time PCR Master Mix (Toyobo, Tokyo, Japan) and specific primers using StepOne plus real-time PCR machine (Applied Biosystems, Carlsbad, CA) as described previously (23). The specific primer sequences and the accession numbers of the target mRNAs are shown in Supplementary Table 1, available at *Carcinogenesis* Online.

### Western blot analysis

Protein extracts from HSC-2 and HSC-3 cells were sonicated on ice in a lysis buffer (20 mM Tris–HCl pH 7.7, 150 mM NaCl, 1 mM ethylenediaminetetraacetic acid, 1% Triton X-100), and were centrifuged at 12,000 rpm for 5 min to remove debris. Then, the protein concentration of the supernatants was determined by Pierce™ BCA protein assay kit (Thermo Fisher Scientific, Rockford, IL, USA) using serially diluted bovine serum albumin as standards. Western blot analysis was performed as described previously (24). The primary antibodies used in this study are shown in Supplementary Table 2, available at *Carcinogenesis* Online.

### Osteoclastogenesis

RAW264.7 cells were cultured in 12- and 24-well plates containing αMEM supplemented with 10% FBS for 24 h. Then, these cells were treated with GST-fused RANKL (GST-RANKL) with or without CCN6 for 2 days. After changing the cultured media, the cells were re-treated with the same factors for another 3 days (22).

### Tartrate-resistant acid phosphatase staining

RAW264.7 cells treated with GST-RANKL were fixed with 4% formaldehyde for 30 min at room temperature, and after being washed with phosphate-buffered saline (PBS), the cells were treated with 0.2% Triton X-100 for 5 min at room temperature. Then, the cells were incubated with 0.01% naphthol AS-MX phosphate (Sigma) and 0.05% fast red violet LB salt (Sigma) in the presence of 50 mM sodium tartrate and 90 mM sodium acetate (pH 5.0). After tartrate-resistant acid phosphatase (TRAP) staining, the cells were washed three times with PBS. For quantitative analysis, TRAP-positive cells with more than three nuclei were counted as osteoclasts (22).

### Gene silencing of CCN6 by siRNA transfection

After HSC-2 cells had reached confluence, the cells were collected with 0.25% trypsin–ethylenediaminetetraacetic acid. Then, 1.0 × 10^6^ cells were re-suspended in OPTI-MEM (Fisher Scientific, Oslo, Norway) containing 10 nM siRNAs against CCN6, which is a pool of three target-specific 19–25-nucleotide siRNAs (Santa Cruz Biotechnology, no. sc-39339) or negative control siRNA, which is a non-targeting 20–25-nucleotide siRNA designed as a negative control (Santa Cruz Biotechnology, no. sc-37007). Electroporation was performed using an NEPA21 (NEPA GENE Co., Ltd., Chiba, Japan) according to the manufacturer’s instructions (23). Thereafter, the cells were cultured for 1 or 2 days until subsequent analyses.

### Indirect immunofluorescence analysis

HSC-3 cells or CCN6 siRNA-treated HSC-2 cells were cultured in non-tissue culture-treated multi-well plates for 1 day. In the case of HSC-3 cells, these cells were treated with BMP2, CCN6 or their combination for 16 h. These CCN6 knocked-down HSC-2 and HSC-3 cells were fixed with 4% formaldehyde for 1 h at room temperature and were made permeable with 0.1% NP-40 in PBS. Indirect immunofluorescence analysis of CCN6 knocked-down HSC-2 and HSC-3 cells was performed with anti-vimentin, anti-E-cadherin and anti-BMP2, anti-CCN6 antibodies (Supplementary Table 2, available at *Carcinogenesis* Online), respectively (25).

### Immunoprecipitation-western blot analysis

After preparation of HSC-2 and HSC-3 cell lysate, rBMP2 (100 µg/ml) at the volume of 5 µl was added into the cell lysate and the cell lysate was incubated at 4°C for 2 h. Then, the cell lysate was reacted with 1 µg of anti-BMP2 antibody at 4°C overnight. For immunoprecipitation, Protein G-Sepharose (GE Healthcare Bio, Uppsala, Sweden) was added and mixed by swirling at 4°C for 4 h. After being washed with PBS three times, the bound proteins were extracted by a sodium dodecyl sulfate sample buffer. Western blot analysis was performed using an anti-CCN6 antibody. Similarly, 0.5 µg CCN6 was added into the 2 µg GST or GST-RANKL solution and was incubated at 4°C overnight. After Glutathione Sepharose (GE Healthcare) beads were reacted for 2 h at room temperature, CCN6 on the beads was detected by western blot using the anti-CCN6 antibody.

### Statistical analysis

All experiments were repeated at least twice, and similar results were obtained. After performing the *F*-test (normality testing), Bonferroni’s test and unpaired Student’s *t*-test were performed to compare the data from multiple groups and from two groups, respectively. All data are presented as the mean values and standard deviations (SDs).

## Results

### Production of CCN family proteins in HSC-2 and HSC-3 cells

**Figure 1.**
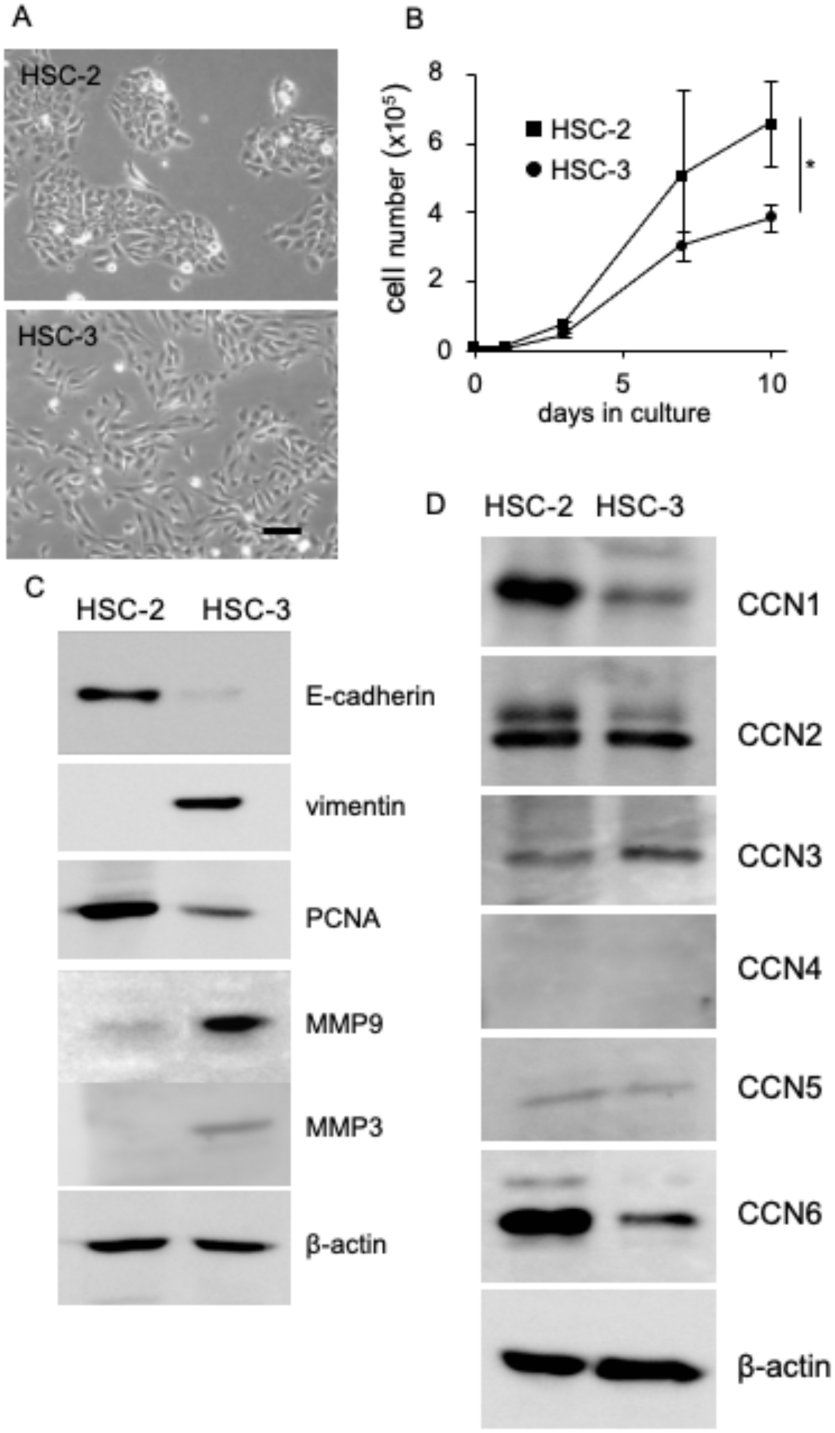
Production of EMT markers and CCN family proteins in HSC-2 and HSC-3 cells. (**A**) Morphological appearance of HSC-2 and HSC-3 cells. HSC-2 and HSC-3 cells were inoculated at a density of 1 × 10^5^ in 35 mm diameter dishes and cultured for 24 h. Phase contrast images of HSC-2 cells and HSC-3 cells are shown. HSC-2 cells grew to form island-like colonies and HSC-3 cells grew to scatter like mesenchymal cells. Bar indicates 100 µm. (**B**) Growth curve of HSC-2 and HSC-3 cells. HSC-2 and HSC-3 cells were inoculated at a density of 1 × 10^5^ cells in 35 mm diameter dishes and cultured with DMEM containing 10% FBS for 10 days. The cell number was counted under microscopy, and the mean and standard deviation were plotted from independent cultures in triplicate. An asterisk indicates a significant difference (*P < 0.05). Western blot analysis of production of EMT markers (**C**) and CCN family proteins (**D**) in HSC-2 and HSC-3 cells. After HSC-2 and HSC-3 cells had reached confluence, the cell lysate was collected, and western blot analysis was performed using the indicated antibodies.

It is well known that both the acquisition of mobility in oral cancer cells and activation of osteoclast formation play important roles in invasion into the jawbone. Therefore, firstly, in order to better explore the molecule regulating both EMT and osteoclastogensis, we focused on CCN family proteins, which have diverse cellular functions, and compared their expression between HSC-2 and HSC-3 cells. As shown in Figure 1A, HSC-2 cells grew to form island-like colonies, which is a characteristic of epithelial cells, whereas HSC-3 cells grew in a scattered manner, like mesenchymal cells. Additionally, the measurement of cell numbers revealed that HSC-2 cells grew significantly better than HSC-3 cells at 10 days after starting cell culture (Figure 1B). Supporting these results, more increased proliferating cell nuclear antigen production was observed in HSC-2 cells than in HSC-3 cells (Figure 1C). Next, we investigated the production of E-cadherin, which is a marker of epithelial cells, and vimentin, which is a marker of mesenchymal cells, using western blot analysis. As shown in Figure 1C, the production of E-cadherin was detected in HSC-2 cells, but not in HSC-3 cells, and vimentin production was detected in HSC-3 cells, but not in HSC-2 cells. Moreover, the production levels of matrix metalloproteinase (MMP)9 and MMP3, which regulate the invasion of oral cancer cells, increased in HSC-3 cells (Figure 1C). These results indicate that HSC-3 cells possess more mesenchymal phenotypes than HSC-2 cells, suggesting that HSC-3 cells acquired high malignancy through EMT. Hence, we hypothesized that HSC-2 cells produce more CCN family members that suppress EMT than HSC-3 cells. As shown in Figure 1D, the productions of CCN1 and CCN6 were higher in HSC-2 cells than in HSC-3 cells. In light of these results and the previous finding that EMT is regulated by WNT signaling (26), we focused on CCN6, which is located downstream of WNT signaling.

### Combination of CCN6 and BMP2 strongly inhibits EMT in HSC-3 cells

**Figure 2.**
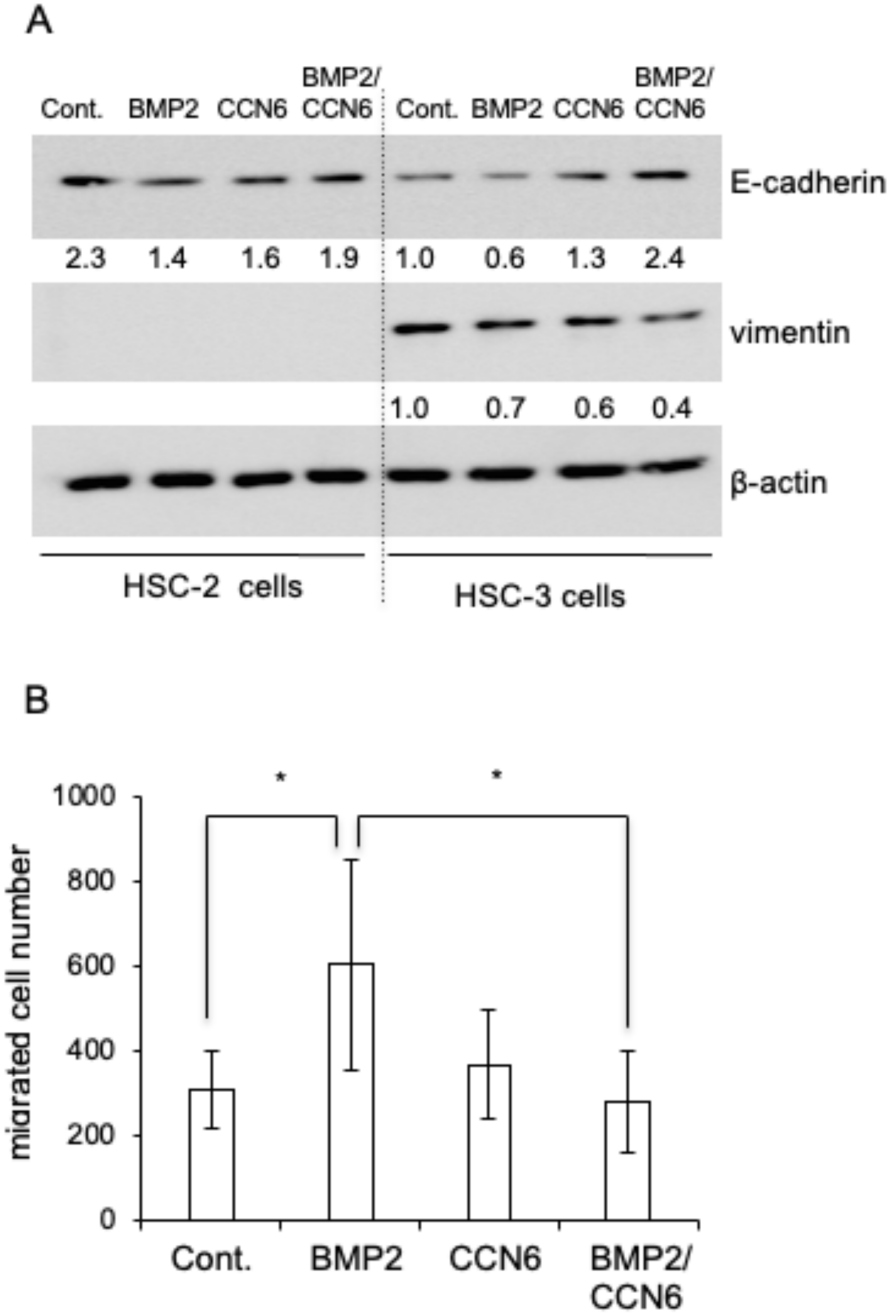
Effect of BMP2, CCN6 or both factors on the production of EMT markers in HSC-2 and HSC-3 cells and on cell migration of HSC-3 cells. (**A**) Western blot analysis of E-cadherin and vimentin production. After HSC-2 and HSC-3 cells had reached confluence, these cells were treated with BMP2 (100 ng/ml), CCN6 (100 ng/ml) or both factors for 48 h. Then, cell lysate was collected, and western blot analysis was performed using anti-E-cadherin, vimentin and β-actin antibodies. The intensities of E-cadherin and vimentin were determined densitometrically, and these intensities were normalized to that of β-actin. Each relative ratio was computed using HSC-3 cells as a control (=1.0). (**B**) Cell migration assay. After CHEMOTAXICELL chambers had been placed on a 24-well plate with 0.3 ml of DMEM containing 10% FBS, HSC-3 cells were inoculated at a density of 1 × 10^4^ cells per 0.2 ml in the same medium. After 4 h, BMP2, CCN6 or both factors were added to the chamber, and the cells were cultured for 20 h. The migrated cells were fixed with 4% formaldehyde and stained with DAPI. The migrated cell number of 4 fields per membrane was counted. The ordinate indicates the sum of 4 fields, and bars represent the mean and standard deviation of 3 sets of experiments with independent culture wells (*n* = 3). Asterisks indicate significant differences, as indicated by the brackets (*P < 0.05). DAPI, 4ʹ,6-diamidino-2-phenylindole.

Next, to investigate whether or not CCN6, BMP2 or both CCN6 and BMP2 regulate EMT in HSC-2 and HSC-3 cells, we stimulated HSC-2 and HSC-3 cells with rCCN6, rBMP2 and both factors. After 24 h, western blot analysis was performed using anti-E-cadherin and anti-vimentin antibodies. Regarding E-cadherin production in HSC-3 cells, when the intensity of the band of the sample treated with the vehicle was represented as 1.0, the intensities of the bands of those treated with CCN6 and BMP2 increased to 1.3 and decreased to 0.6, respectively (Figure 2A). Interestingly, the intensity of the band from the sample treated with both CCN6 and BMP2 exhibited the greatest increase, reaching 2.4 (Figure 2A). In contrast, the production of vimentin in the cells treated with CCN6 combined with BMP2 showed the greatest decrease (Figure 2A). In HSC-2 cells, the intensity of the band of E-cadherin following treatment with BMP2 was lower than that following vehicle treatment, but those with CCN6 and CCN6 combined with BMP2 were comparable to that with the vehicle (Figure 2A). Vimentin production was not detected in HSC-2 cells. Next, we investigated cell migration of HSC-3 cells treated with rCCN6, rBMP2 or CCN6 combined with BMP2, using a modified Boyden chamber method. As shown in Figure 2B, cell migration was significantly promoted by rBMP2, and BMP2-promoted cell migration was significantly suppressed by the co-treatment with CCN6. These findings suggest that CCN6 inhibits EMT of HSC-3 cells in the presence of BMP2.

### CCN6 knockdown promotes EMT in HSC-2 cells

**Figure 3.**
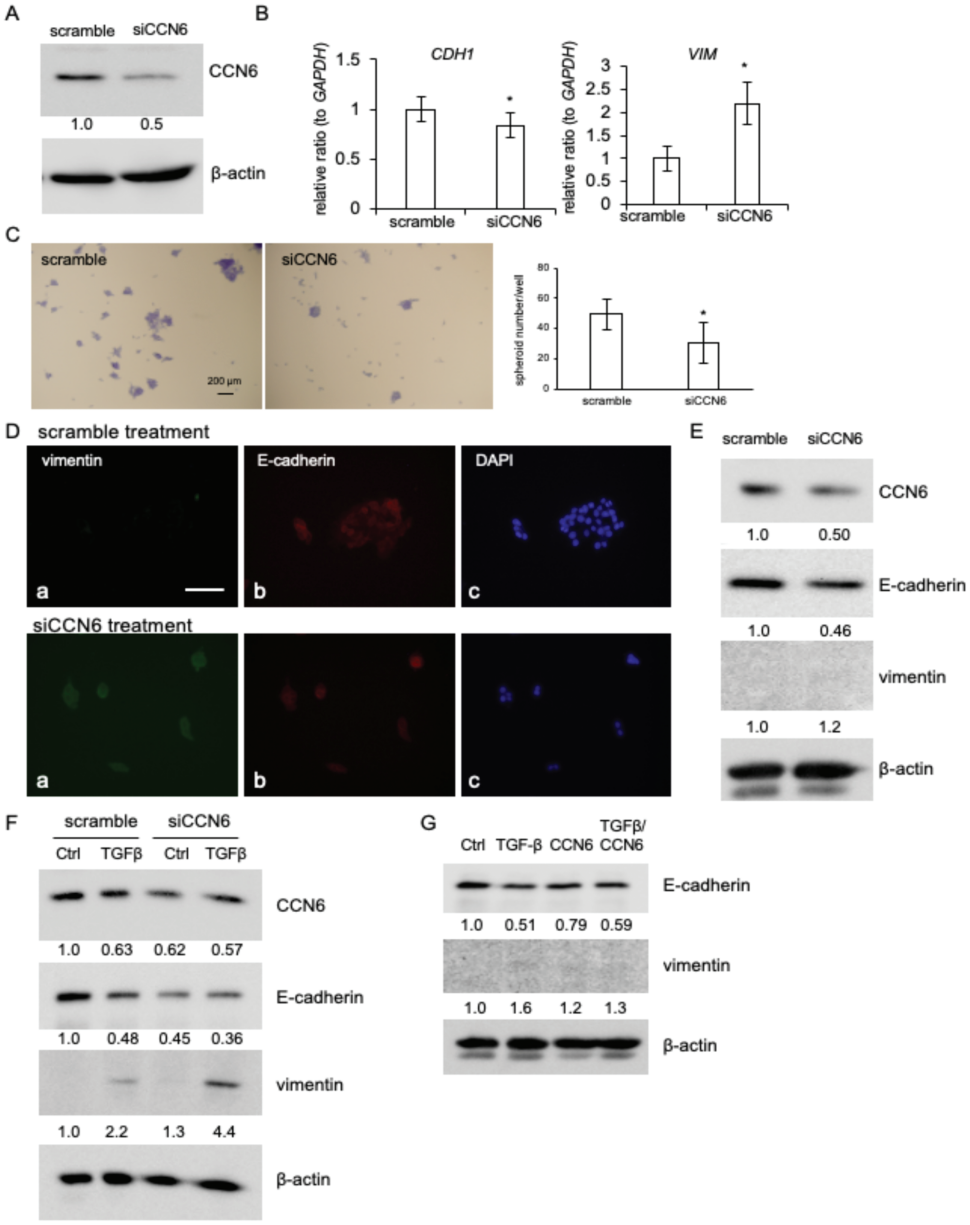
Effect of CCN6 knockdown on E-cadherin and vimentin expression in HSC-2 cells. (**A**) Western blot analysis of CCN6 production. HSC-2 cells were transfected with siRNA against CCN6 (siCCN6) or scramble siRNA (final concentrations: 10 nM) by using electroporation, and 48 h later, western blot analysis was performed. The amount of CCN6 was determined densitometrically, and this amount was normalized to the amount of β-actin. Relative ratio was calculated against the value of scramble treatment (=1.0). (**B**) Gene expression of E-cadherin (*CDH1*) and vimentin (*VIM*). HSC-2 cells were treated with siCCN6, and total RNA was collected after 24 h. Quantitative RT–PCR analysis was performed using specific primers for *CDH1* and *VIM*. The amounts of these mRNAs were normalized to the amount of *GAPDH* mRNA. The bars represent mean and standard deviation data from independent culture dishes (*n* = 9), which was analyzed by Student’s *t*-test. Asterisks indicate significant differences (*P < 0.05). (**C**) Quantification of the spheroid number. After HSC-2 cells were treated with siCCN6, these cells were inoculated into non-tissue culture-treated multi-well plates and cultured for 24 h. Then, toluidine blue staining was performed (left panel; representative images, bar represents 200 µm), and spheroids in 5 fields per well were counted. The ordinate indicates the sum of 5 fields, and bars represent the mean and standard deviation of 5 wells. Asterisks indicate significant differences (*P < 0.05). (**D**) Indirect immunofluorescence analysis of vimentin and E-cadherin. After the culture of HSC-2 cells treated with siCCN6 was inoculated into non-tissue culture-treated multi-well plates and kept for 24 h, indirect immunofluorescence analysis was performed with anti-vimentin and anti-E-cadherin antibodies. Vimentin was detected in the spheroids formed by CCN6 knocked-down HSC-2 cells but was not in the spheroids with scramble siRNA (panel ‘a’). E-cadherin was detected in both scramble and siCCN6-treated cells (panel ‘b’). Bars represent 50 µm. (**E**) Western blot analysis of CCN6, E-cadherin and vimentin in spheroid-formed HSC-2 cells. CCN6 knocked-down HSC-2 cells were cultured under the same condition as (D), and the cell lysate was collected. Then, western blot analysis was performed. The intensities of CCN6, E-cadherin and vimentin signals were determined densitometrically, and these intensities were normalized to that of β-actin. Each relative ratio was standardized against the scrambled control (scramble = 1.0). (**F**) Effect of CCN6 knockdown on TGF-β-induced EMT in HSC-2 cells. After treatment with siCCN6 for 24 h, the HSC-2 cells were stimulated by TGF-β (10 ng/ml) for 24 h. Then, western blot analysis was performed, and the signal intensities of described proteins were determined. These intensities were normalized to those of β-actin. Each relative ratio was computed by using scrambled sample without TGF-β as a control (=1.0). (**G**) Effect of TGF-β, CCN6 or both factors on EMT markers in A431 cells. After A431 cells had reached confluence, these cells were treated with TGF-β (10 ng/ml), CCN6 (100 ng/ml) or both factors for 48 h. Then, western blot analysis was performed, and the signal intensities of described proteins were determined. These intensities were normalized to those of β-actin. Each relative ratio was computed by using vehicle treatment as a control (=1.0).

To confirm that CCN6-by itself suppresses EMT, we treated HSC-2 cells, which produce more CCN6 than HSC-3 cells, with siRNA against CCN6 for 48 h. After confirming knockdown of CCN6 (Figure 3A), we investigated the gene expression levels of E-cadherin and vimentin using real-time reverse-transcription (RT)–PCR analysis. As shown in Figure 3B, E-cadherin and vimentin expression levels decreased and increased in CCN6 knocked-down HSC-2 cells compared with those treated with scramble siRNA, respectively. Moreover, we examined the effect of CCN6 knocked-down in HSC-2 cells on spheroid formation, which mimics the *in vivo* behavior of cancer cells. It was observed that spheroid formation was repressed in CCN6 knocked-down HSC-2 cells compared with those with scrambled controls (Figure 3C, left panel; spheroids stained with toluidine blue, right graph; quantified data of spheroid number per well). Additionally, indirect immunofluorescence analysis revealed that the production of vimentin was detected in CCN6 knocked-down cells, but not in scrambled controls, and E-cadherin was detected in both CCN6 knocked-down and scramble siRNA-treated cells (Figure 3D). To support these data, we performed western blot analysis to detect E-cadherin and vimentin production in spheroid-formed HSC-2 cells treated with siRNA against CCN6 or scramble siRNA. As shown in Figure 3E, knockdown of CCN6 showed a decrease and increase in the production of E-cadherin and vimentin, respectively. These findings indicate that downregulation of CCN6 promotes EMT, suggesting that CCN6-by itself negatively regulates EMT in oral cancer cells. Next, to further confirm the inhibitory effect of CCN6 on EMT, western blot analysis was performed with CCN6 knocked-down HSC-2 cells treated with TGF-β, which strongly promotes EMT. As shown in Figure 3F, TGF-β treatment decreased and increased E-cadherin and vimentin production in scrambled controls, respectively. On the other hand, in CCN6 knocked-down cells, vimentin production was synergistically increased (Figure 3F). In contrast, CCN6 did not affect EMT in epidermoid carcinoma A431 cells, which are not oral cancer cells, stimulated by TGF-β (Figure 3G). These results suggest that CCN6 regulates BMP2- and TGF-β-induced EMT specifically in oral cancer cells.

### CCN6 suppresses RANKL-induced osteoclastogenesis

**Figure 4.**
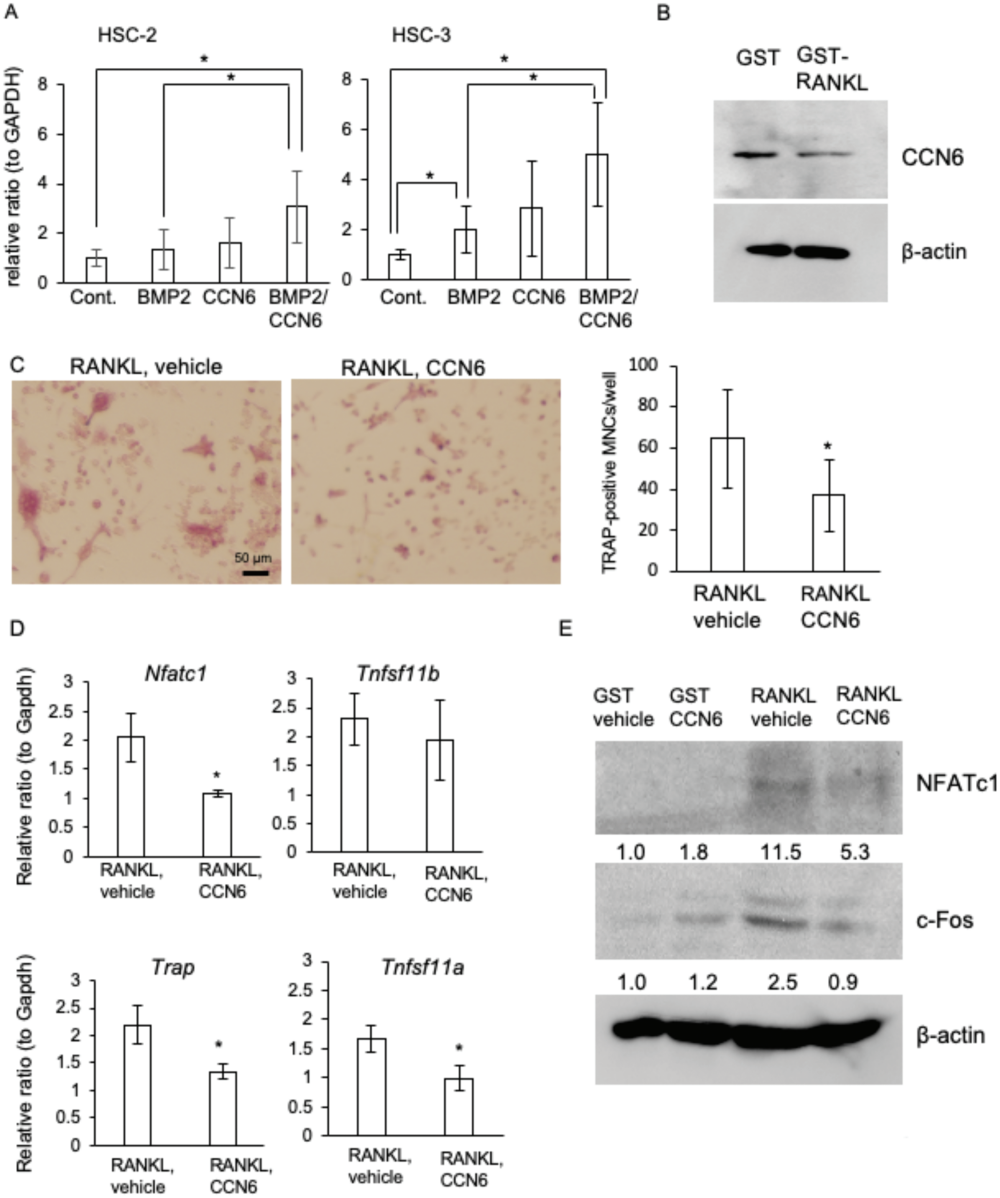
Effect of CCN6 on the differentiation of RAW264.7 cells into osteoclast-like cells. (**A**) Gene expression of *TNFSF11* (RANKL) in HSC-2 and HSC-3 cells treated with BMP2, CCN6 or both factors. After these cells had reached confluence, the cells were treated with BMP2, CCN6 or both factors for 24 h. The amounts of the mRNAs were normalized to that of *GAPDH* mRNA. The ordinate indicates the relative ratio with respect to vehicle treatment (ratio = 1.0), and bars represent mean and standard deviation values of 3 sets in the experiments of triplicate cultures. Asterisks indicate significant differences, as indicated by the bars (*P < 0.05). (**B**) CCN6 production in the differentiated multi-nuclear giant RAW264.7 cells. RAW264.7 cells were treated with 200 µg/ml GST-RANKL for 6 days and differentiated into osteoclast-like cells. Western blot analysis was performed using anti-CCN6 and β-actin antibodies. (**C**) TRAP staining in RAW264.7 cells treated with CCN6 combined with GST-RANKL and the quantification of TRAP-positive multinucleated cells (MNCs). RAW264.7 cells were treated with GST-RANKL in the presence and absence of 100 ng/ml CCN6 for 5 days, and the cells were stained with TRAP (left panel; representative images, bar represents 50 µm). Then, TRAP-positive MNCs of 6 fields per well were counted. The ordinate indicates the sum of 6 fields, and bars represent the mean and standard deviation of the data from 6 wells (right graph). Asterisks indicate significant differences (*P < 0.05). (**D**) Gene expression of osteoclastic markers in RAW264.7 cells treated with GST-RANKL and CCN6. RAW264.7 cells were treated with GST-RANKL in the absence and presence of CCN6 for 7 days, and total RNA was collected. Quantitative RT–PCR analysis was performed using specific primers. The amounts of the mRNAs were normalized to that of *Gapdh* mRNA. The bars represent the mean and standard deviation of triplicate cultures. Asterisks indicate significant differences (*P < 0.05). (**E**) Western blot analysis of NFATc1 and c-Fos production in RAW264.7 cells treated with GST-RANKL and CCN6. RAW264.7 cells were treated with GST or GST-RANKL in the presence and absence of CCN6 for 7 days, and cell lysate was collected. Then, western blot analysis was performed using anti-NFATc1, c-Fos and β-actin antibodies. Relative band intensities against each control (GST-vehicle) are also shown.

Next, to investigate the effect of the combination of CCN6 and BMP2 on osteoclast formation, which is essential for bone metastasis, we investigated the gene expression of RANKL, which plays important roles in osteoclast formation, in HSC-2 and HSC-3 cells treated with CCN6 and BMP2. As shown in Figure 4A, RANKL expression was upregulated more by the treatment with the CCN6 combined with BMP2 than with the treatment with vehicle or BMP2 alone in HSC-2 and HSC-3 cells. Interestingly, RANKL expression was significantly upregulated by BMP2 alone in HSC-3 cells, but not in HSC-2 cells (Figure 4A). These results indicate that CCN6 and BMP2 cooperatively upregulate RANKL expression. Therefore, we next examined the effect of CCN6 on RANKL-induced osteoclast formation. When RAW264.7 cells, which are osteoclast progenitor cells, were stimulated by GST-RANKL for 5 days, the cells differentiated into multi-nuclear giant cells that were positive for TRAP staining (Supplementary Figure 1, available at *Carcinogenesis* Online). At this time point, the differentiated multi-nuclear giant RAW264.7 cells decreased CCN6 production (Figure 4B). Surprisingly, the number of TRAP-positive RAW264.7 cells with more than three nuclei decreased significantly following treatment with both CCN6 and RANKL, compared with following treatment with RANKL alone (Figure 4C). Additionally, quantitative RT–PCR analysis revealed that the gene expression levels of osteoclast differentiation markers, such as *Nfatc1*, *Trap* and *Rank*, decreased following treatment with both CCN6 and RANKL, compared with those with RANKL alone (Figure 4D). In support of these results, the protein production of osteoclast differentiation markers, such as NFATc1 and c-Fos, was also decreased following treatment with both CCN6 and RANKL (Figure 4E). These findings suggest that CCN6 suppresses osteoclast formation induced by RANKL.

### CCN6 interacts with BMP2 and RANKL

**Figure 5.**
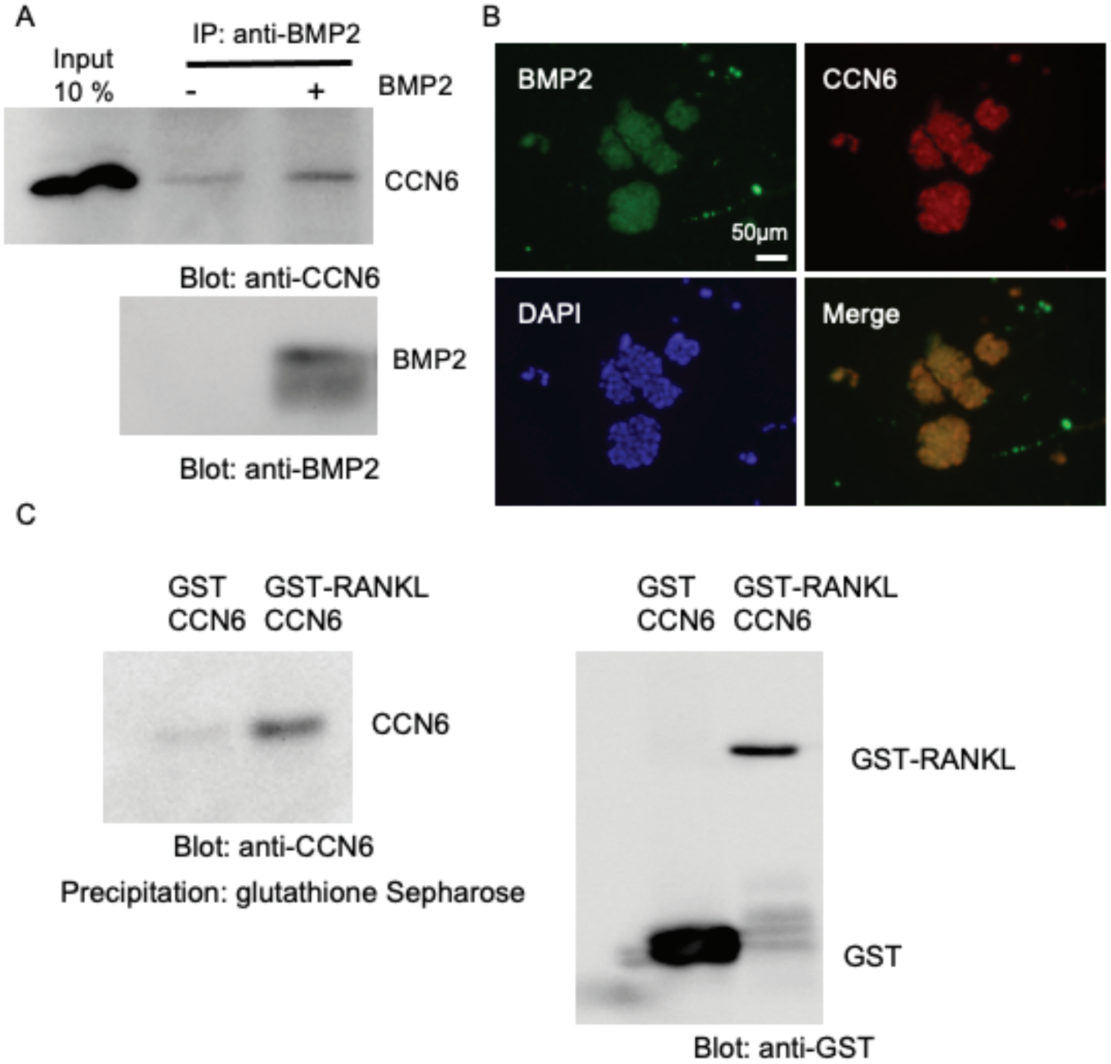
Interaction and co-localization of CCN6 and BMP2, and interaction of CCN6 with RANKL. (**A**) IP-western blot analysis of CCN6 and BMP2. After HSC-2 cells had reached confluence, cell lysate was collected. Then, the cell lysate was combined with or without 1 µg BMP2 and incubated at 4°C for 2 h. The cell lysate was treated with 1 µg anti-BMP2 antibody at 4°C overnight, and BMP2 and associated proteins were precipitated with protein G-Sepharose. Western blot analysis was performed using anti-CCN6 and anti-BMP2 antibodies. When BMP2 was added into the solution, CCN6 was clearly detected. (**B**) Indirect immunofluorescence analysis of CCN6 and BMP2. HSC-3 cells were inoculated in non-tissue culture-treated multi-well plates and cultured for 24 h. Then, indirect immunofluorescence analysis was performed with anti-CCN6 and anti-BMP2 antibodies. CCN6 and BMP2 were co-localized in the spheroids formed by HSC-3 cells. (**C**) Interaction of CCN6 with GST-RANKL. Recombinant CCN6 (0.5 µg) was combined with 2 µg GST or GST-RANKL and incubated at 4°C overnight. Then, Glutathione-Sepharose was added and reacted for 2 h at room temperature. After washing with PBS three times, western blot analysis of the bound proteins was performed using anti-CCN6 and anti-GST antibodies. When GST-RANKL was added into the solution, CCN6 was detected. IP, immunoprecipitation.

Next, we examined whether or not CCN6 interacted with BMP2 and RANKL. To investigate the interaction with BMP2, we prepared cell lysate from HSC-2 cells, which produced large amounts of CCN6. After performing immunoprecipitation using an anti-BMP2 antibody in the presence or absence of rBMP2, western blot analysis was performed using an anti-CCN6 antibody. As shown in Figure 5A, the band of CCN6 increased in the presence of rBMP2, and the band of added rBMP2 was also detected by using the anti-BMP2 antibody. We also investigated interaction of CCN6 with BMP2 using HSC-3 cell lysate, which produced less amounts of CCN6. As a result, the band of CCN6 was perceivable in the complex immunoprecipitated from HSC-3 cell lysate by the anti-BMP2 antibody (Supplementary Figure 2, available at *Carcinogenesis* Online). These results indicate that CCN6 interacts with BMP2 *in vitro*. Next, to analyze the localization of BMP2 and CCN6 *in vivo*, we performed immunofluorescence analysis using the spheroid formation of HSC-3 cells. As shown in Figure 5B, CCN6 and BMP2 were co-localized in all spheroids. These results suggest that CCN6 interacts with BMP2 *in vivo* structures of cancer cells. Next, we examined the interaction of CCN6 with RANKL *in vitro*. After rCCN6 protein and GST-fused RANKL were reacted on ice for 2 h, the complexes were precipitated with Glutathione Sepharose. Then, CCN6 in the precipitated sample was detected using the anti-CCN6 antibody. As a result, CCN6 was detected in the complex with GST-fused RANKL, but not detected in the complex with GST (Figure 5C). These results indicate that CCN6 interacts with RANKL as well.

### Combination of CCN6 with BMP2 enhances Smad1/5/9 and p38 signaling pathways

**Figure 6.**
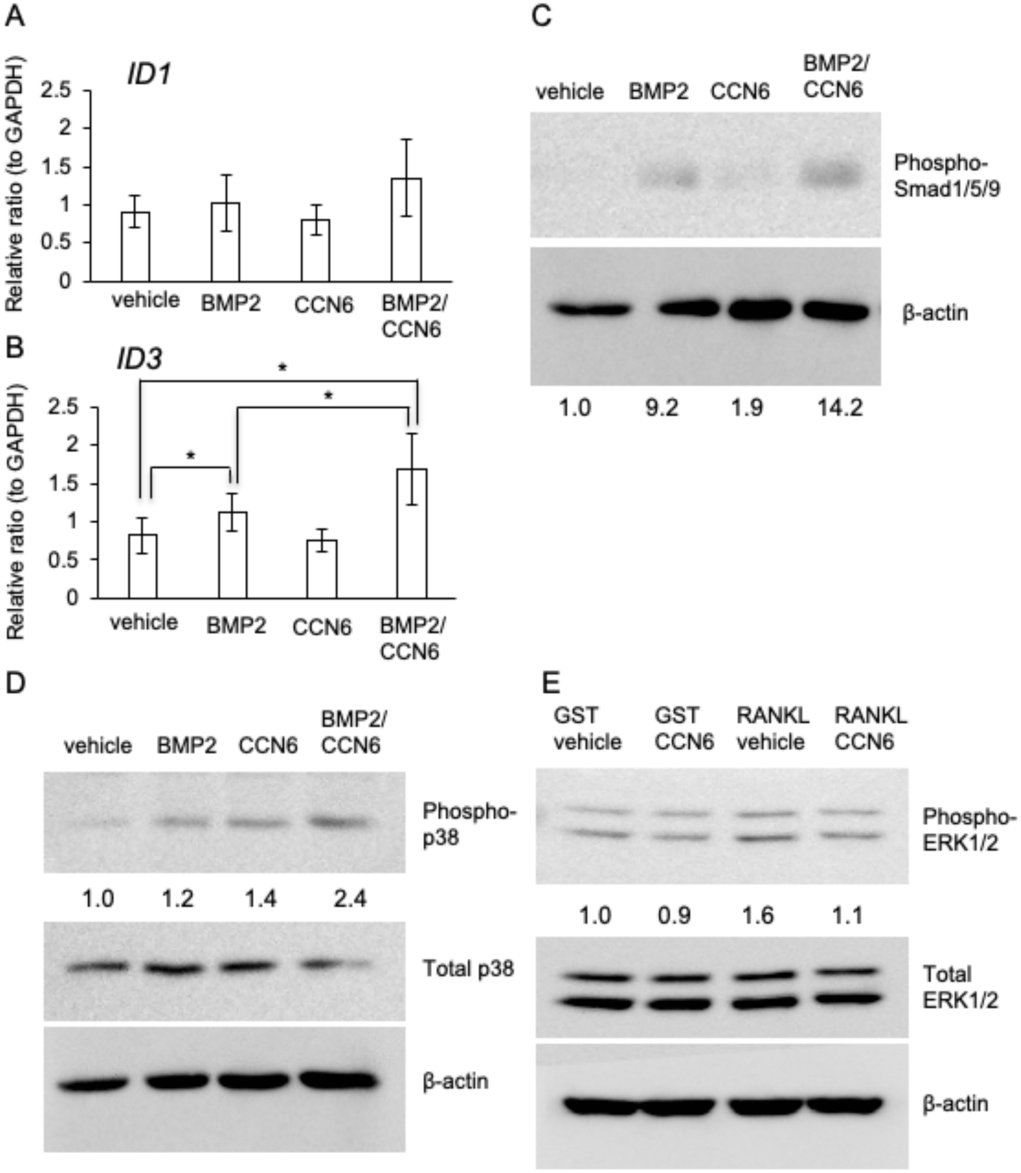
Effect of CCN6 combined with BMP2 on Smad and p38 signaling pathways in HSC-3 cells and effect of CCN6 combined with RANKL on ERK1/2 phosphorylation in RAW264.7 cells. (**A** and **B**) Gene expression of ID1 and ID3 in HSC-3 cells treated with BMP2, CCN6 or both factors. After HSC-3 cells had reached confluence, the cells were treated with BMP2, CCN6 or CCN6 combined with BMP2 for 18 h. Then, total RNA was collected, and quantitative RT–PCR analysis was performed using specific primers for ID1 (A) and ID3 (B). The amounts of these mRNAs were normalized to that of *GAPDH* mRNA. The bars represent the mean and standard deviation of the data from independent culture dishes (*n* = 12). Asterisks indicate significant differences (*P < 0.05). Western blot analysis of Smad (**C**) and p38 (**D**) phosphorylation in HSC-3 cells treated with BMP2, CCN6 or both factors. After HSC-3 cells had reached confluence, these cells were treated with BMP2, CCN6 or both factors for 30 min. Then, cell lysate was collected and western blot analysis was performed using the indicated specific antibodies. The intensities of the bands of phospho-Smad1/5/9 and phospho-p38 were determined densitometrically, and these intensities were normalized to that of β-actin. Each relative ratio was computed against the control (vehicle treatment = 1.0). (**E**) Western blot analysis of ERK1/2 phosphorylation in RAW264.7 cells treated with GST-RANKL, CCN6 or both factors. RAW264.7 cells were inoculated in 35 mm diameter dishes. The following day, the cells were treated with GST or GST-RANKL for 30 min in the absence and presence of CCN6. Then, cell lysate was collected and western blot analysis was performed using anti-phospho-ERK1/2, total ERK1/2 and β-actin antibodies. The intensity of the band of phospho-ERK1/2 was determined densitometrically, and this intensity was normalized to that of total ERK. Each relative ratio was standardized against the control (vehicle treatment = 1.0).

As our data revealed that CCN6 interacted with BMP2, we next investigated whether or not CCN6 combined with BMP2 modulated the Smad1/5/9 and p38 signaling pathways in HSC-3 cells. Because it was known that inhibitor of DNA binding 1 (ID1) and ID3 are downstream transcriptional targets of both Smad and p38 signaling pathways in multiple cell types (27), we examined the gene expression of ID1 and ID3 in HSC-3 cells treated with BMP2, CCN6 or both factors. Although ID1 expression had no effect (Figure 6A), the gene expression of ID3 was significantly increased in response to BMP2, and it increased even more following treatment with the combination of CCN6 and BMP2 than BMP2 alone (Figure 6B). Moreover, consistent with the result shown in Figure 6B, phosphorylation of Smad1/5/9 was enhanced by treatment with BMP2 alone, and it was enhanced more by the treatment with CCN6 combined with BMP2 (Figure 6C). Similarity to Smad1/5/9, phosphorylation of p38 was increased more by CCN6 combined with BMP2 than by BMP2 or CCN6 alone (Figure 6D). These results indicate that CCN6 forms a complex with BMP2 and enhances the phosphorylation of Smad1/5/9 and p38, thus suggesting that CCN6 positively modulates BMP signaling pathways.

### Combination of CCN6 with RANKL suppresses ERK1/2 activation

Next, we investigated whether or not combination of CCN6 with RANKL modulated the phosphorylation of ERK1/2 induced by RANKL in RAW264.7 cells. As shown in Figure 6E, CCN6 that interacted with RANKL suppressed phosphorylation of ERK1/2 induced by RANKL alone. These findings suggest that CCN6 also modulates the RANKL signaling pathway, but in a negative manner, in contrast to BMP signaling pathways.

## Discussion

In this study, we found that CCN6 interacted with BMP2, and their combination increased E-cadherin production and decreased vimentin production. Additionally, we also showed that CCN6 interacted with RANKL, and their combination suppressed osteoclastogenesis of RAW264.7 cells. These results indicate that CCN6 suppresses EMT and osteoclast formation via interaction with BMP2 and RANKL, respectively, suggesting that CCN6 suppresses bone invasion/metastasis of oral cancer cells. In fact, our data revealed that CCN6 combined with BMP2 suppressed BMP2-induced cell migration of HSC-3 cells (Figure 2B). It was previously reported that CCN6 suppresses breast cancer metastasis by binding to BMP4 and blocking BMP4-mediated activation of p38 (20). Moreover, it was also shown that CCN6 decreases EMT and invasion by attenuating IGF-1 receptor signaling in breast cancer (19). On the basis of these reports, CCN6 has been considered a suppressor of breast cancer invasion and metastasis. Similarly, our data showed that CCN6 production was lower in HSC-3 cells with high malignancy, than in HSC-2 cells with low malignancy, and that knockdown of CCN6 decreased the gene expression and protein production of E-cadherin and increased those of vimentin in HSC-2 cells. Moreover, knockdown of CCN6 enhanced EMT stimulated by TGF-β (Figure 3F). These findings indicate that CCN6 suppresses EMT, suggesting that CCN6 serves as a suppressor of tumor invasion in OSCC cells. Therefore, we hypothesized that CCN6 antagonized BMP signaling via binding to BMP2, as was observed in breast cancer cells. To verify this hypothesis, we evaluated the gene expression levels of *ID1* and *ID3*, which are downstream transcriptional targets of both Smad1/5/9 and p38 signaling pathways (27), in HSC-3 cells treated with BMP2 alone or combination of CCN6 and BMP2 by real-time RT–PCR analysis. As a result, *ID1* expression had no effect; however, *ID3* expression was unexpectedly upregulated by the stimulation with combination of CCN6 and BMP2, compared with that with BMP2 alone (Figure 6A and B). Additionally, this finding is supported by results showing that the combination of CCN6 and BMP2 increased the phosphorylation of Smad1/5/9 and p38, compared with BMP2 or CCN6 alone (Figure 6C and D). These results also indicate that CCN6 combined with BMP2 enhances not only the BMP signaling pathway, but also the p38 pathway. Our data showed that cell migration of HSC-3 cells increased with BMP2 treatment (Figure 2B) and that BMP2 as well as CCN6 production was higher in HSC-2 cells without mesenchymal phenotype than that in HSC-3 cells (Supplementary Figure 3, available at *Carcinogenesis* Online). These findings indicate that increased BMP2 and CCN6 productions in oral cancer cells before EMT suppress invasion, and after EMT, BMP2 promotes cell migration due to the absence of CCN6. Therefore, BMP2 yields different biological outcomes, depending upon the presence or absence of CCN6. This notion is also supported by reports indicating that BMP2 induces EMT (28), or other reports indicating that BMP2 induces mesenchymal–epithelial transition (MET) (29). Moreover, it has also been reported that ID3 overexpression suppresses the invasion of squamous cell carcinoma cells (30). Considering that ID3 expression, which is a target gene of both Smad1/5/9 and p38, was increased by CCN6 combined with BMP2, and that production of CCN6 and BMP2 increased in the cells before EMT, it is suggested that CCN6 combined with BMP2 suppresses EMT of oral cancer cells in primary lesions. However, further investigation is needed to uncover the detailed mechanism of EMT suppression by CCN6 with BMP2 in OSCC cells.

It is well known that osteoclasts are involved in bone destruction associated with cancer cells (31). Generally, osteoclast formation and bone resorption are regulated via the RANK/RANKL signaling system during bone remodeling. Accordingly, the key molecule in the mechanism of cancer-associated bone destruction is RANKL produced by cancer cells (31). In fact, production of RANKL by tumor cells has been reported in breast (32), prostate (33) and oral cancers (34), and the RANKL produced by oral cancer cells has been reported to actually participate in cancer-associated bone destruction (34). In this study, we showed that the gene expression level of RANKL was upregulated with BMP2 treatment in HSC-3 cells, and this upregulation was enhanced more by CCN6 combined with BMP2 than by BMP2 alone in HSC-2 and HSC-3 cells (Figure 4A). These findings suggest that CCN6 combined with BMP2 plays an important role in bone resorption associated with oral cancer cells. On the other hand, our data revealed that CCN6 interacted with RANKL and that CCN6 combined with RANKL suppressed the differentiation to osteoclasts of RAW264.7 cells (Figure 4C–E). These findings indicate that CCN6 suppresses osteoclast formation from osteoclast precursor cells via binding to RANKL, suggesting that CCN6 has an effect similar to osteoprotegerin. Regarding human skeletal disorders, more than 20 mutations in CCN6 have so far been identified in patients with progressive pseudorheumatoid dysplasia, which is a genetic bone disorder characterized by severe osteoarthritis and osteopenia of spine (35,36). However, the role of CCN6 in progressive pseudorheumatoid dysplasia phenotypes remains unknown because both CCN6 deficient and CCN6 overexpressed mouse models were histologically indistinguishable from the wild-type (37). Considering the results of the present study indicating that CCN6 suppresses osteoclast formation by inhibiting the RANKL signaling pathway, the phenotype of osteopenia seen in patients with progressive pseudorheumatoid dysplasia may at least in part be due to the excessive osteoclast formation by loss-of-function mutations in CCN6.

On the basis of the results reported here, we developed a working model for the dual roles of CCN6 in OSCC in primary and metastatic lesions. In primary lesions, OSCC cells acquire mobility via EMT induced by several factors, including TGF-β and BMP2, and the cell migration of OSCC cells showing mesenchymal phenotypes by EMT is promoted by BMP2. Then, OSCC cells reach the metastatic lesions, such as in the jawbone, through blood or lymphatic vessels. In metastatic lesions, these OSCC cells convert to cells with epithelial phenotypes via MET. These cells produce more CCN6 than the cells with mesenchymal phenotypes. On the other hand, BMP2 is released from bone matrices destroyed by osteoclasts, and CCN6 and BMP2 form a complex. CCN6 combined with BMP2 promotes MET of newly migrated OSCC cells as well as production of RANKL. Although CCN6 could suppress osteoclast formation via binding with RANKL, interaction of CCN6 and RANKL is interfered with by the interaction of CCN6 with BMP2. Therefore, osteoclast formation is promoted by RANKL produced from OSCC cells, and these osteoclasts further release BMP2 and provide metastasis space for OSCC cells by destroying the bone matrix. As a result, CCN6 combined with BMP2 strongly promotes the metastasis of OSCC cells into the bone tissues.

In conclusion, our results show that CCN6 binds to BMP2 and RANKL and that CCN6 combined with BMP2 suppresses EMT, namely, by promoting MET. Although CCN6 combined with RANKL suppresses osteoclastogenesis, this effect can be nullified by BMP2. These data suggest the dual roles of CCN6 in the invasion and metastasis of OSCC cells in primary and metastatic lesions. Our data provide evidence that CCN6 could be a therapeutic target for the development of treatments to suppress the settlement and invasion of oral cancer cells in the jawbone.

## Supplementary Material

bgad057_suppl_Supplementary_Figure_S1

bgad057_suppl_Supplementary_Figure_S2

bgad057_suppl_Supplementary_Figure_S3

bgad057_suppl_Supplementary_Tables

## Data Availability

All data supporting the findings of this study can be found in the article and in its supplementary information. The data are available from the corresponding author upon responsible request.

## Abbreviations

BMP2: bone morphogenetic protein 2
CCN6: cellular communication network factor 6
EMT: epithelial–mesenchymal transition
GST: glutathione *S*-transferase
HSC-2: human squamous carcinoma-2
HSC-3: human squamous carcinoma-3
ID3: inhibitor of DNA binding 3
MET: mesenchymal–epithelial transition
OSCC: oral squamous cell carcinoma
RANKL: receptor activator of nuclear factor-κB ligand
