# Supplementary figures and images for: Dual roles of cellular communication network factor 6 (CCN6) in the invasion and metastasis of oral cancer cells to bone via binding to BMP2 and RANKL
Source: Carcinogenesis. 2023 Aug 17;44(8-9):695–707. doi: 10.1093/carcin/bgad057 (PMC10692700; doi:10.1093/carcin/bgad057)

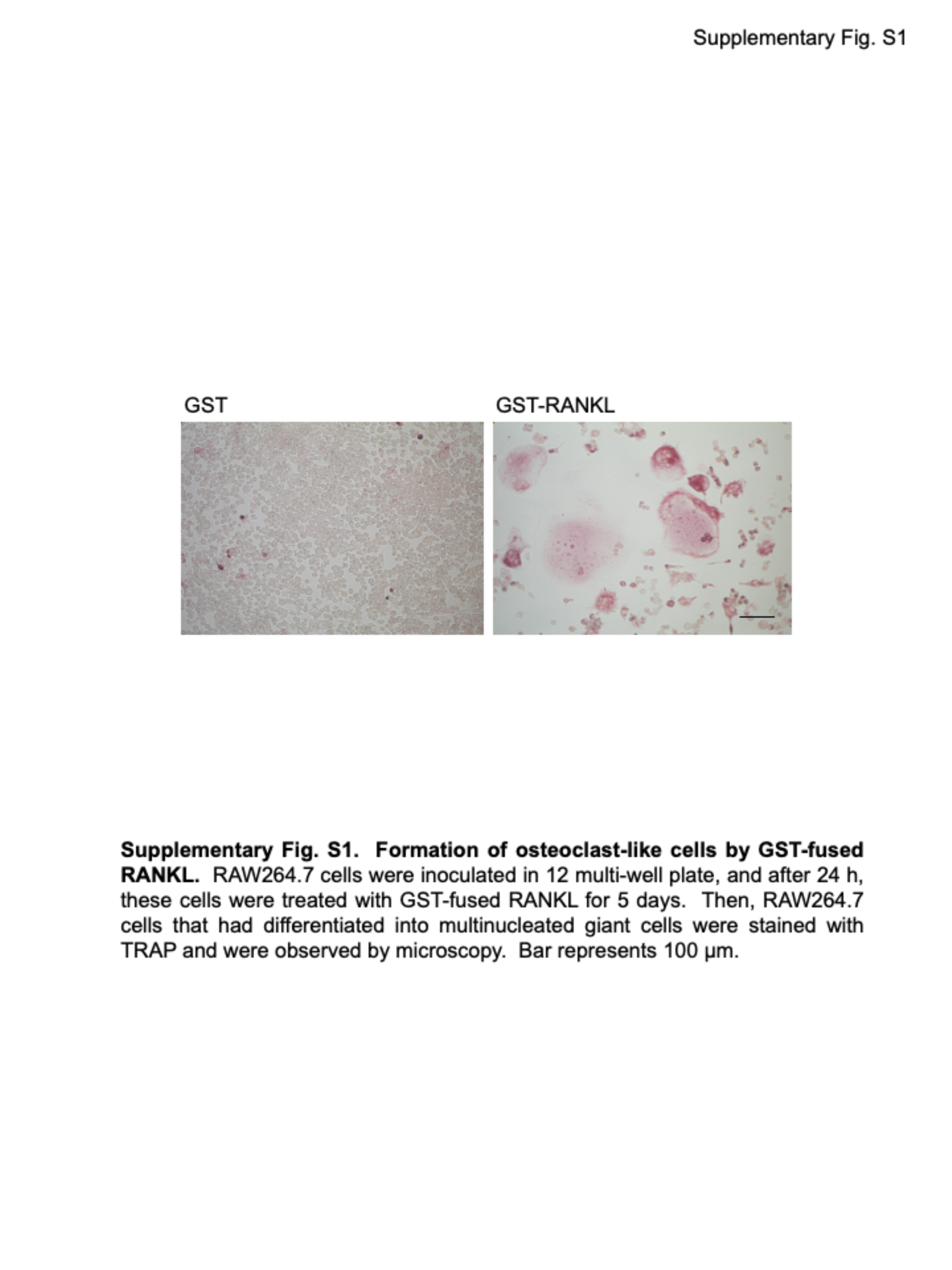

Supplement: bgad057_suppl_Supplementary_Figure_S1 [file bgad057_suppl_supplementary_figure_s1.jpeg]

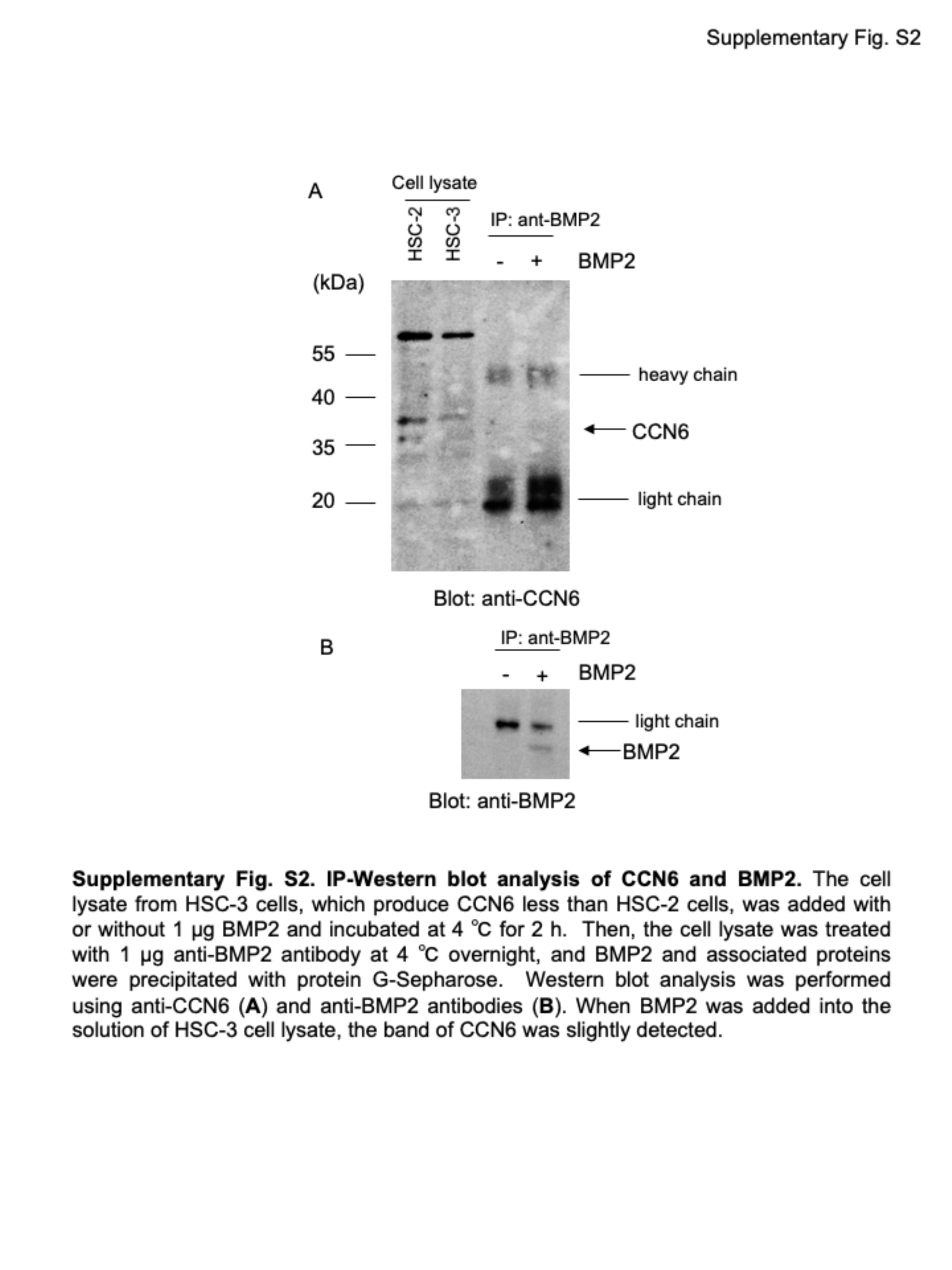

Supplement: bgad057_suppl_Supplementary_Figure_S2 [file bgad057_suppl_supplementary_figure_s2.jpeg]

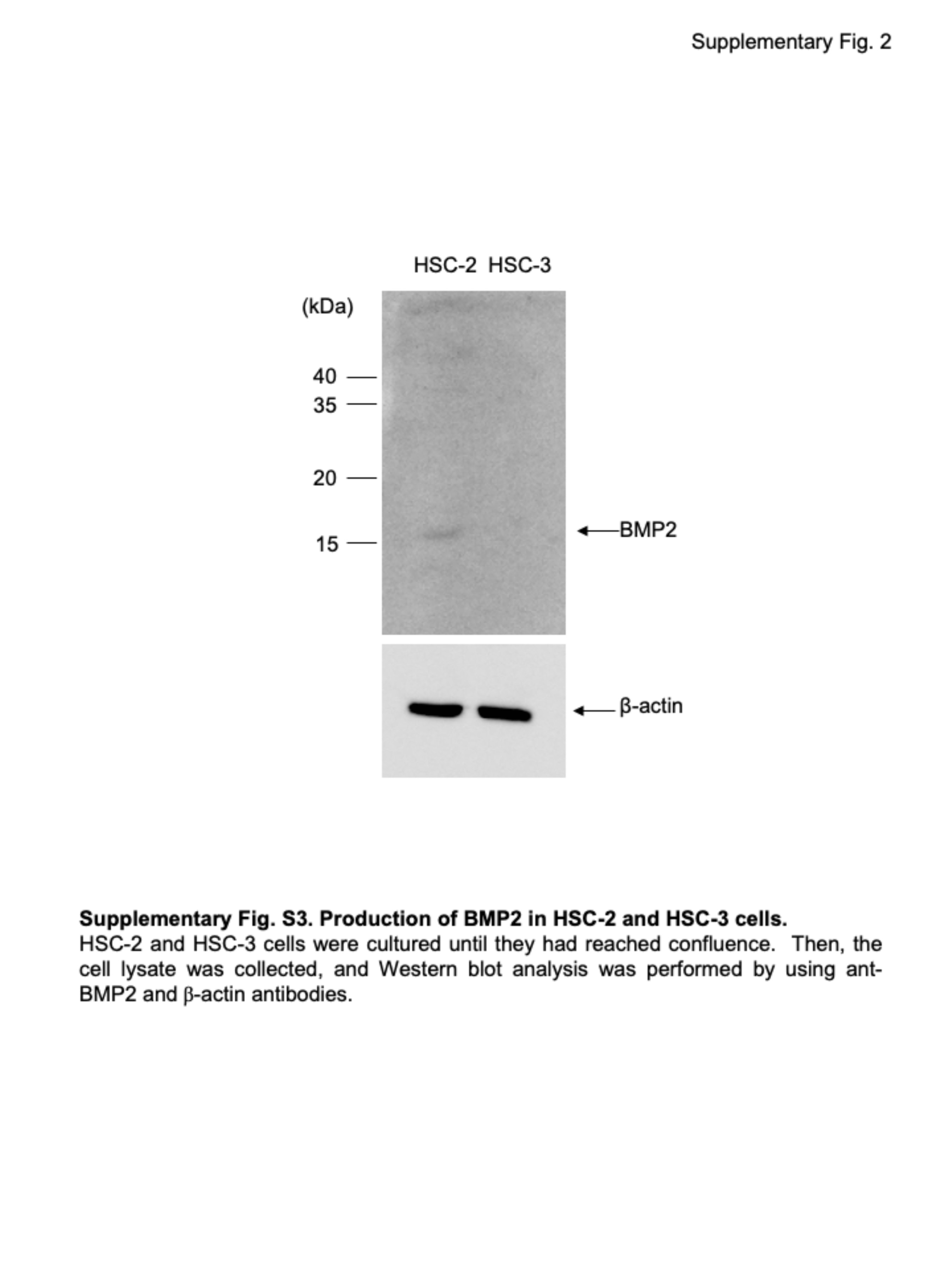

Supplement: bgad057_suppl_Supplementary_Figure_S3 [file bgad057_suppl_supplementary_figure_s3.jpeg]
