# Supplementary material for: Dual roles of cellular communication network factor 6 (CCN6) in the invasion and metastasis of oral cancer cells to bone via binding to BMP2 and RANKL
Source: Carcinogenesis. 2023 Aug 17;44(8-9):695–707. doi: 10.1093/carcin/bgad057 (PMC10692700; doi:10.1093/carcin/bgad057)
Supplement: bgad057_suppl_Supplementary_Tables [file bgad057_suppl_supplementary_tables.docx]

| Gene | Accession no. | species | Nucleotide Sequence |
| --- | --- | --- | --- |
| *CDH1*  *(E-cadherin)* | NM_004360 | human | (F) 5’-TACACTGCCCAGGAGCCAGA-3’  (R) 5’-TGGCACCAGTGTCCGGATTA-3’ |
| *VIM*  *(VIMENTN)* | NM_003380.5 | human | (F) 5’-GGTGGACCAGCTAACCAACGA-3’  (R) 5’-TCAAGGTCAAGACGTGCCAGA-3’ |
| *ID1* | NM_002165.4 | human | (F)5’-GGTGGAGATTCTCCAGCACG-3’ (R) 5’-TCCAACTGAAGGTCCCTGATG-3’ |
| *ID3* | NM_002167.5 | human | (F) 5’-TTCCCATCCAGACAGCCG -3’  (R) 5’-GCGTTCTGGAGGTGTCAGGA 3’ |
| *TNFSF11*  *(RANKL)* | NM_003701.4 | human | (F) 5’-ACTGGATCCGGATCAGGATG-3’  (R) 5’-AGCTGCGAAGGAGCACATGA-3’ |
| *GAPDH* | XM_011241214.1 | human/  mouse | (F) 5’-GCCAAAAGGGTCATCATCTC-3’  (R) 5’-GTCTTCTGGGTGGCAGTGAT-3’ |
| *Nfatc1* | NM_001164112.1 | mouse | (F) 5’-TGAGGCTGGTCTTCCGAGTT-3’  (R )5’-CGCTGGGAACACTCGATAGG-3’ |
| *Trap* | NM_007388.3 | mouse | (F) 5’-CGACCATTGTTAGCCACATACG-3  (R) 5’-TCGTCCTGAAGATACTGCAGGTT-3’ |
| *Tnfrsf11b*  *(Opg)* | NM_008764.4 | mouse | (F) 5’-AGCTGCTGAAGCTGTGGAA-3’  (R) 5’-AGGTTCGAGTGGCCGAGAT-3’ |
| *Tnfrsf11a*  *(RANK)* | NM_009399.5 | mouse | (F) 5’-TGCCTACAGCATGGGCTTT-3’  (R) 5’-AGAGATGAACGTGGAGTTACTGTTT-3’ |

Supplementary Table 1.

Sequences of forward (F) and reverse (R) primers used for quantitative PCR

| Primary antibody | Makers | Catalog no. | Dilution  WB IF |
| --- | --- | --- | --- |
| CCN1 | Santa-Cruz | sc-13100 | 1:100 |
| CCN2 | Abcam | ab66992 | 1:1000 |
| CCN3 | CST | 8767S | 1:1000 |
| CCN4 | Santa-Cruz | sc-25441 | 1:100 |
| CCN5 | Santa-Cruz | sc-25442 | 1:100 |
| CCN6 | Santa-Cruz | sc-25443 | 1:100 1:100 |
| BMP2 | Santa-Cruz | sc-6895 | 1:100 1:100 |
| E-cadherin | CST | 24E10 | 1:1000 1:500 |
| vimentin | American Research Product | VIM3B4 | 1:1000 1:500 |
| MMP9 | Triple Point Biologics, Inc. | RP1-MMP-9 | 1:1000 |
| MMP3 | Santa-Cruz | sc-6839 | 1:100 |
| PCNA | Sigma | p-8825 | 1:000 |
| NFATc1 | Santa-Cruz | sc-7294 | 1:100 |
| c-Fos | CST | 2250S | 1:1000 |
| GST | MERCK | 27-4577-01 | 1:1000 |
| Phospo-Smad1/5/9 | CST | 13820S | 1:1000 |
| Smad1/5/8 | Santa-Cruz | sc-6031-R | 1:100 |
| Phospo-p38 | CST | 4511S | 1:1000 |
| Total p38 | CST | 9212 | 1:1000 |
| β-actin | Sigma | 010-27841 | 1:1000 |

Supplementary Table 2.

List of primary antibodies used by Western blot analysis and immunofluorescence analysis
